# Supplementary material for: Amino acids bind to phase-separating proteins and modulate biomolecular condensate stability and dynamics
Source: Nat Commun. 2025 Sep 30;16:8723. doi: 10.1038/s41467-025-63755-x (PMC12484591; doi:10.1038/s41467-025-63755-x)
Supplement: Supplementary file 1 — Supplementary Information [file 41467_2025_63755_MOESM1_ESM.pdf]

Supplementary Information

Amino acids bind to phase-separating proteins and modulate biomolecular condensate stability and dynamics

Xufeng Xu[a]*, Merlijn H. I. van Haren[a], Iris B. A. Smokers[a], Brent S. Visser[a], Paul B. White[a], Robert S. Jansen[b], Evan Spruijt[a]*

[a] Institute for Molecules and Materials, Radboud University, Nijmegen, The Netherlands
[b] Department of Microbiology, Radboud Institute for Biological and Environmental Sciences, Radboud University, Nijmegen, The Netherlands
*Correspondence: evan.spruijt@ru.nl; xfengxu@hotmail.com

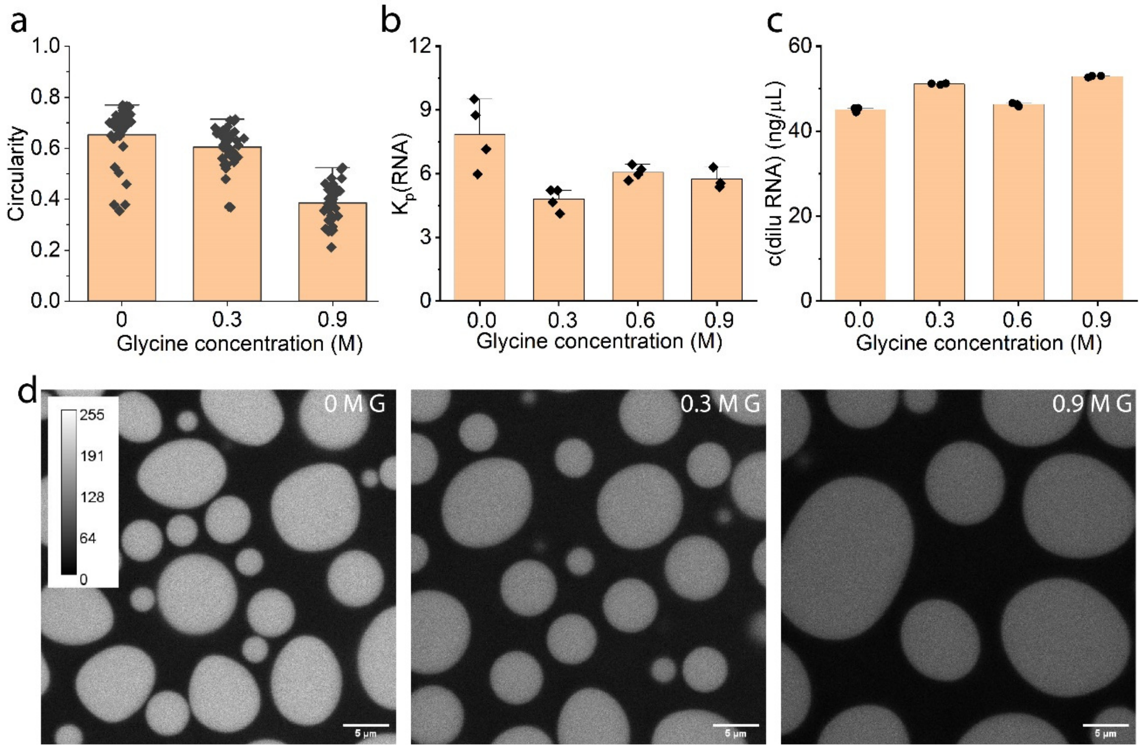

Supplementary Figure 1: (a) Condensate circularity in Figure 1b, using the particle analysis function in FIJI, (b) Partition coefficient and (c) concentration in the dilute phase of RNA for NPM1-RNA condensates as a function of glycine concentration, (d) Confocal fluorescence microscopy images of NPM1-RNA condensates in RP3 channel after the addition of 0, 0.3, and 0.9 M glycine (laser power: 50%, the color bar on the left). Scale bar = 5 μm. Data are expressed as mean ± standard deviation of n ≥ 3 independent measurements.

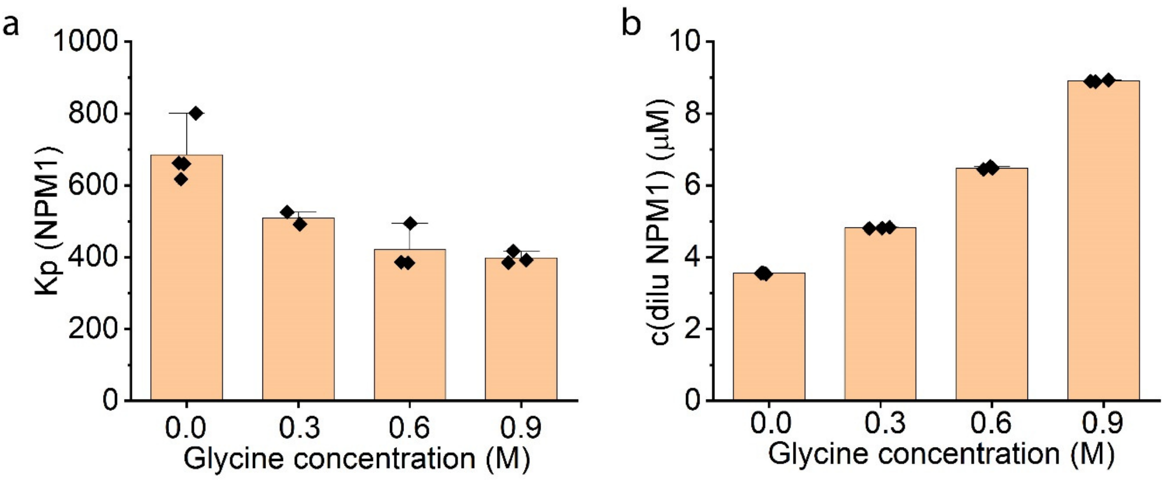

Supplementary Figure 2: (a) Partition coefficient and (b) concentration in the dilute phase of NPM1 for NPM1 condensates in the presence of PEG (10 kDa) crowding as a function of glycine concentration. Data are expressed as mean ± standard deviation of n = 3 independent experiments.

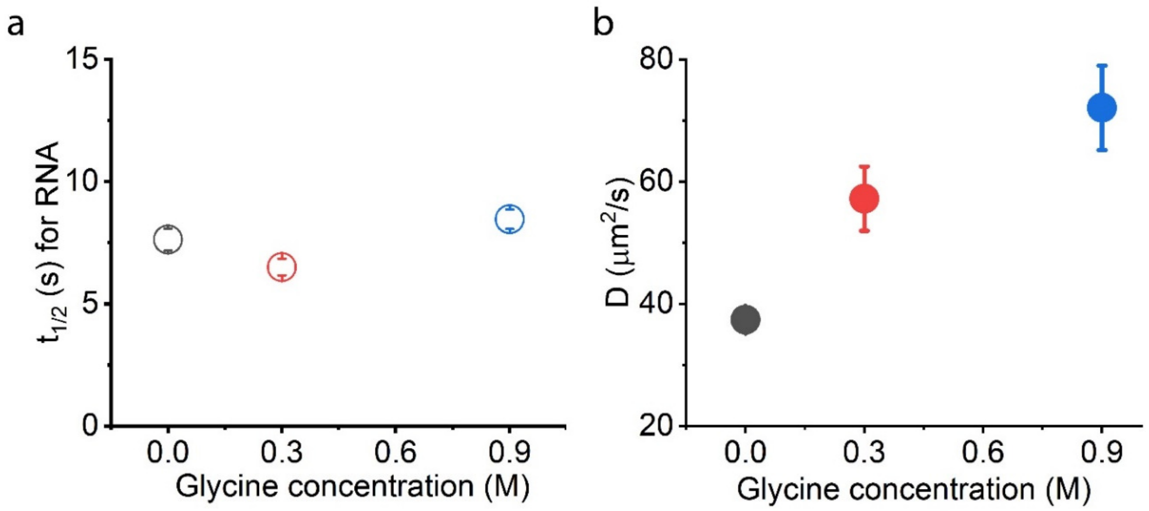

Supplementary Figure 3: (a) Recovery half-life ($t_{1/2}$) of RNA after bleaching as a function of glycine concentration and (b)Diffusion coefficient (D) of fluorescein (Alexa Fluor 488 or A488) in NPM1-RNA condensates after the addition of glycine (0, 0.3, and 0.9 M), calculated by fitting the autocorrelation curves of A488. Data are expressed as mean ± standard deviation of n = 3 independent experiments.

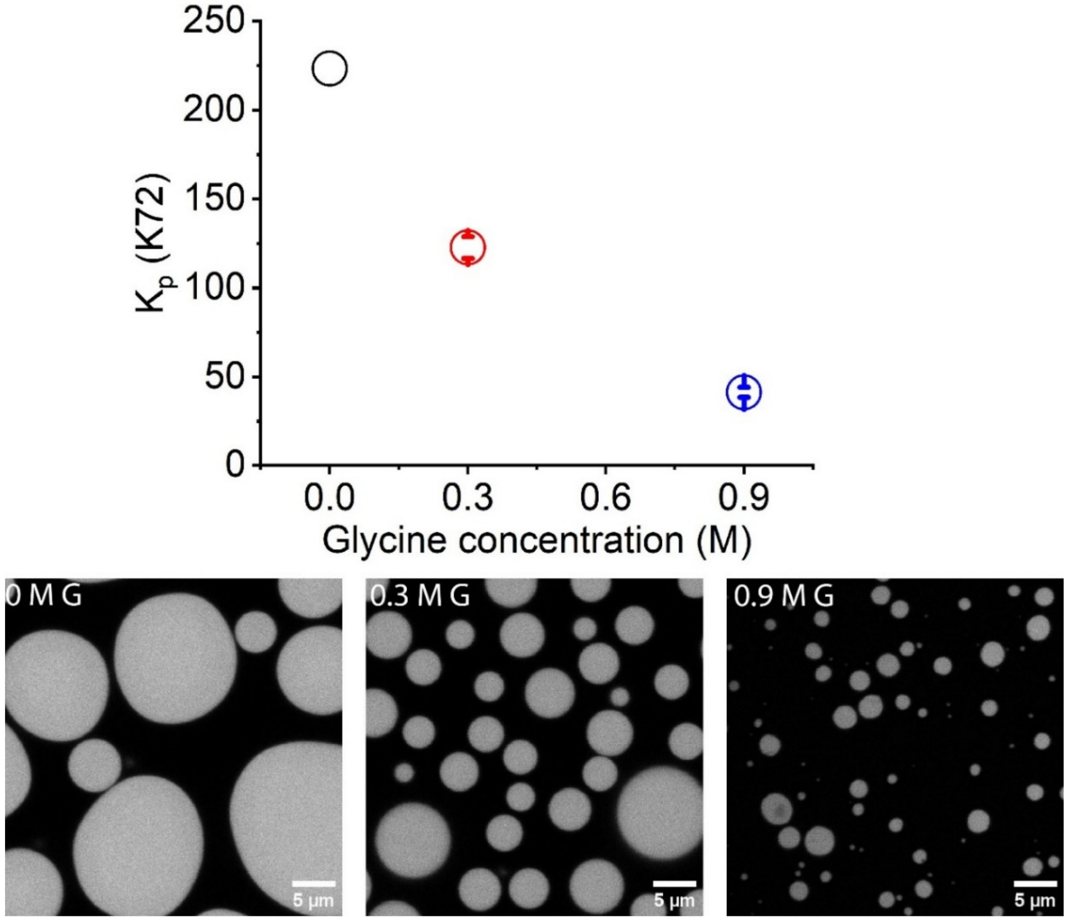

Supplementary Figure 4: Partition coefficient of K72 in the condensate phase for K72-ATP condensates as a function of glycine concentration and representative confocal fluorescence microscopy images of the condensates in K72 channel. Data are expressed as mean ± standard deviation of n = 3 independent experiments. Scale bar = 5 µm.

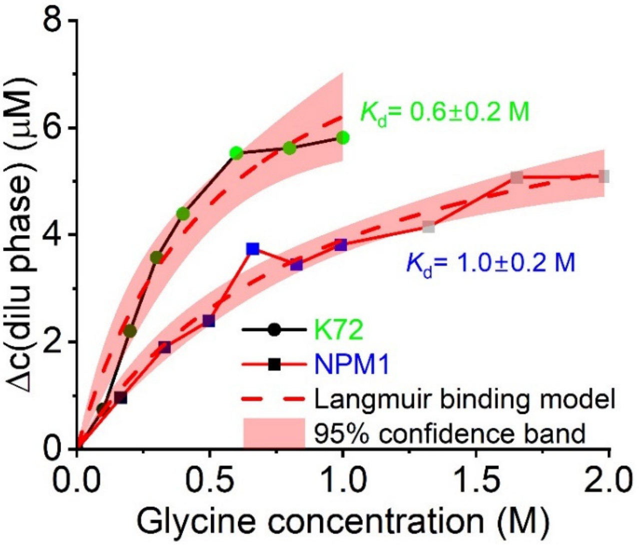

Supplementary Figure 5: Effect of glycine on the protein/peptide concentration in the dilute phase for NPM1-RNA (red curve) and K72-ATP (black curve) condensates. Data are expressed from n = 1 independent experiments. The fitting curves are in red dashed lines with a 95% confidence band (red) using the Langmuir-type binding model.

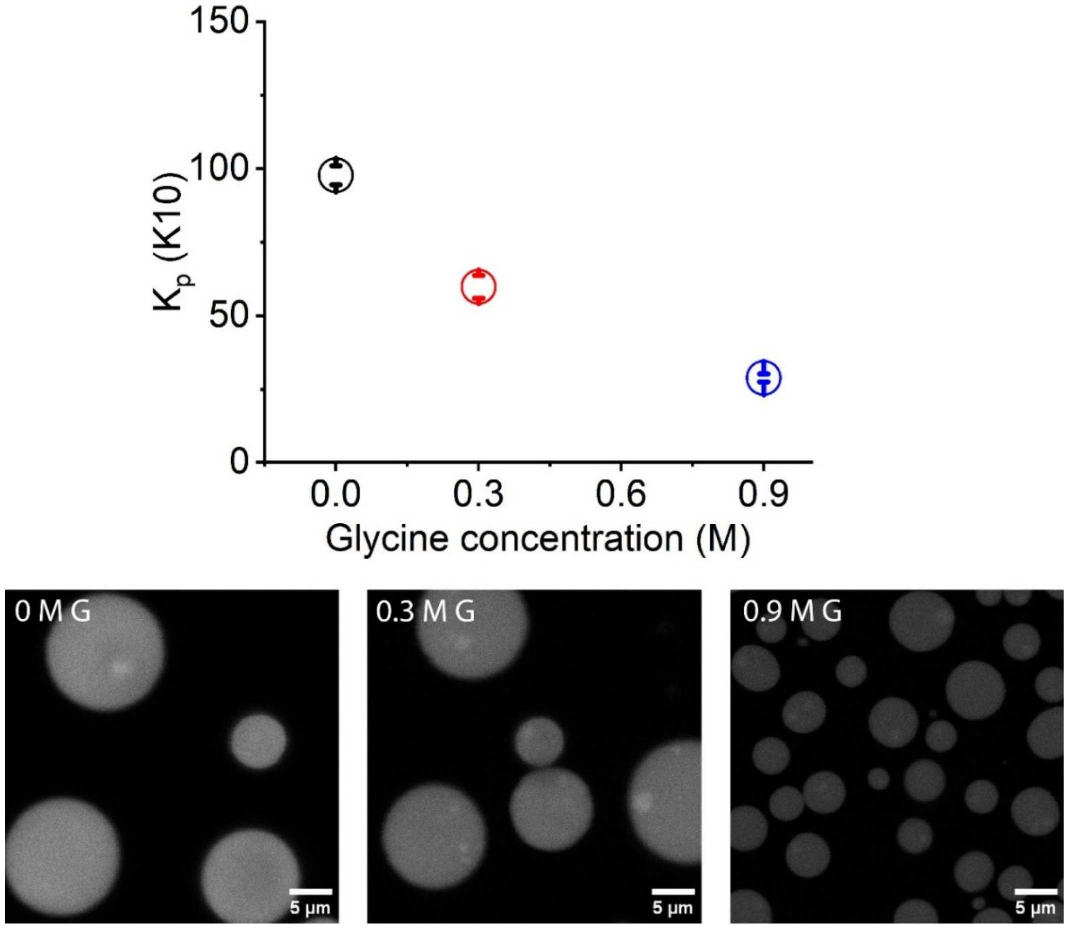

Supplementary Figure 6: Partition coefficient of K10 in the condensate phase for K10-D10 condensates as a function of glycine concentration and representative confocal fluorescence microscopy images of the condensates in K10 channel. Data are expressed as mean ± standard deviation of n = 4 independent experiments.

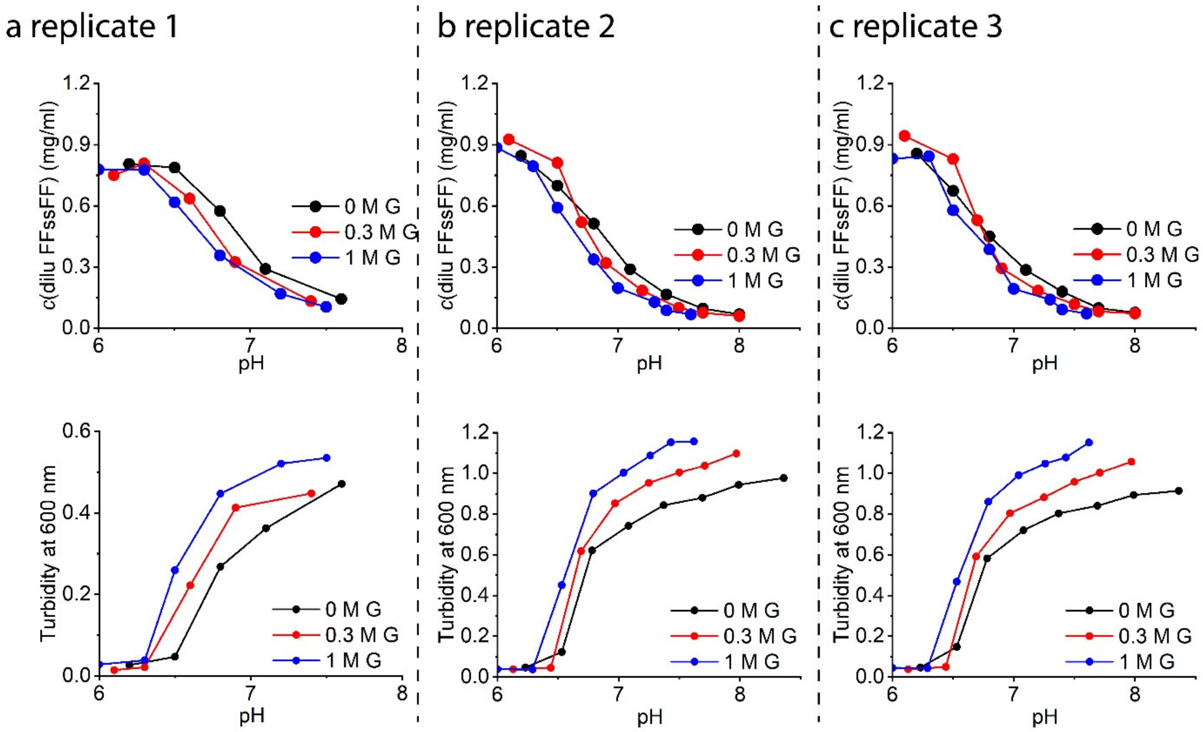

Supplementary Figure 7: Three replicates of the FFssFF concentration in the dilute phase and the turbidity of FFssFF at pH from 6 to 8 at different glycine concentrations (0, 0.3, and 1 M). Data are from n = 3 independent experiments.

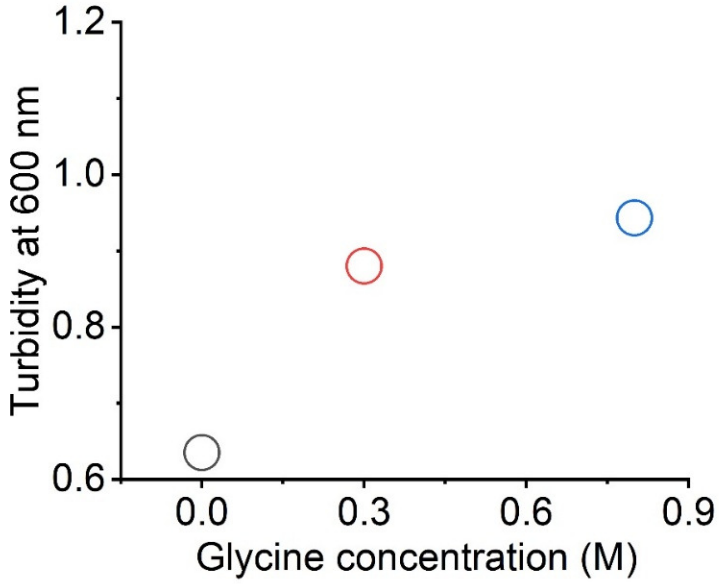

Supplementary Figure 8: Turbidity of WGR-4 at different glycine concentrations (0, 0.3, and 0.8 M). Data are expressed from n = 1 independent experiments.

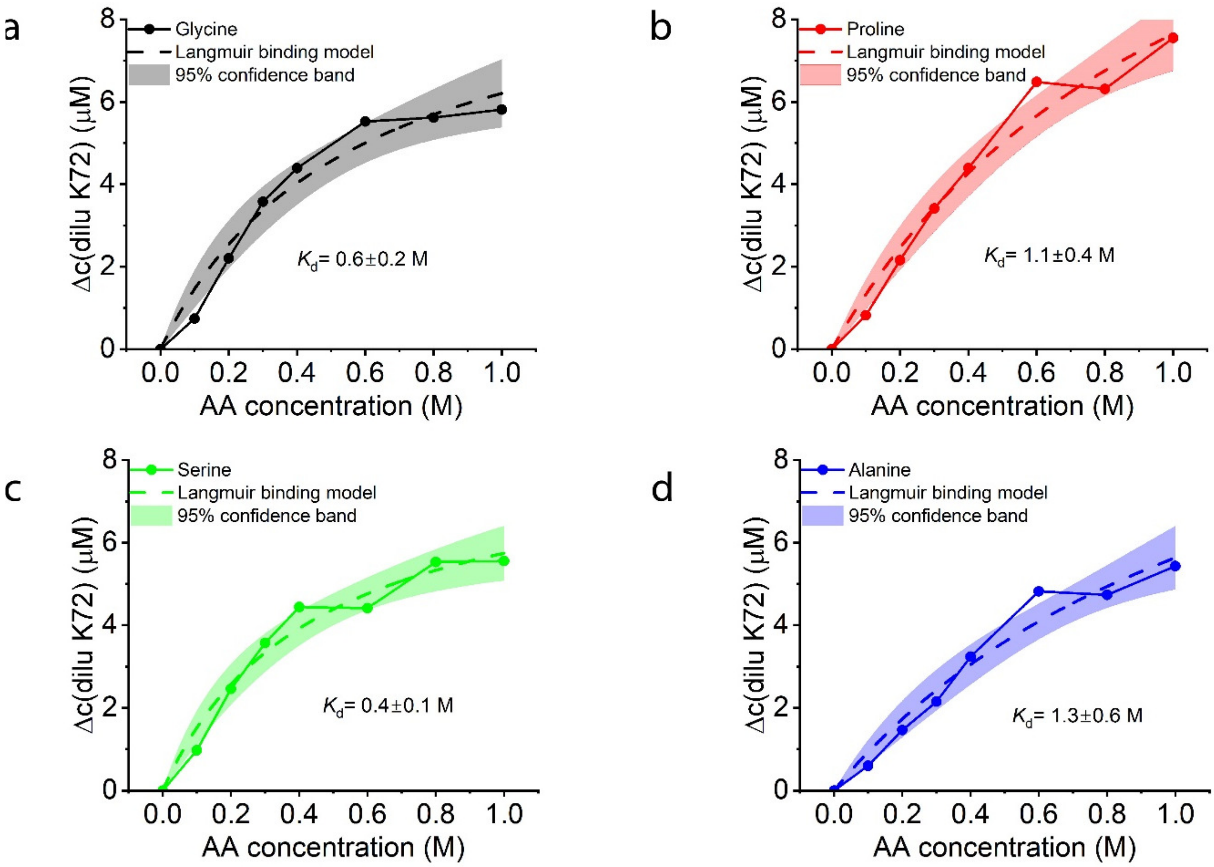

Supplementary Figure 9: K72 concentration in the dilute phase for K72-ATP condensates as a function of the concentration of four representative AAs, including (a) glycine; (b) proline; (c) serine; and (d) alanine. The fitting curves are in dashed lines with a 95% confidence band using the Langmuir-type binding model. Data are expressed from n = 1 independent experiments.

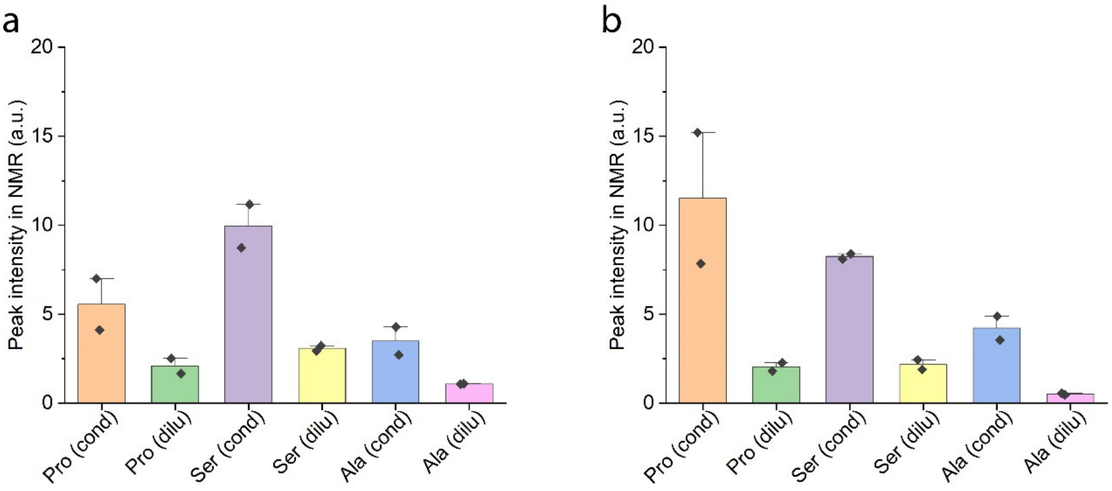

Supplementary Figure 10: NMR peak intensity raw data for amino acid concentrations in condensate and dilute phases for (a) NPM1-RNA condensates and (b) K72-ATP. *K*p for glycine is not possible to obtain in ^{1}H NMR as its peak is hidden under background buffer peaks at around 3.5 ppm. Data are expressed as mean ± standard deviation of n = 2 independent experiments.

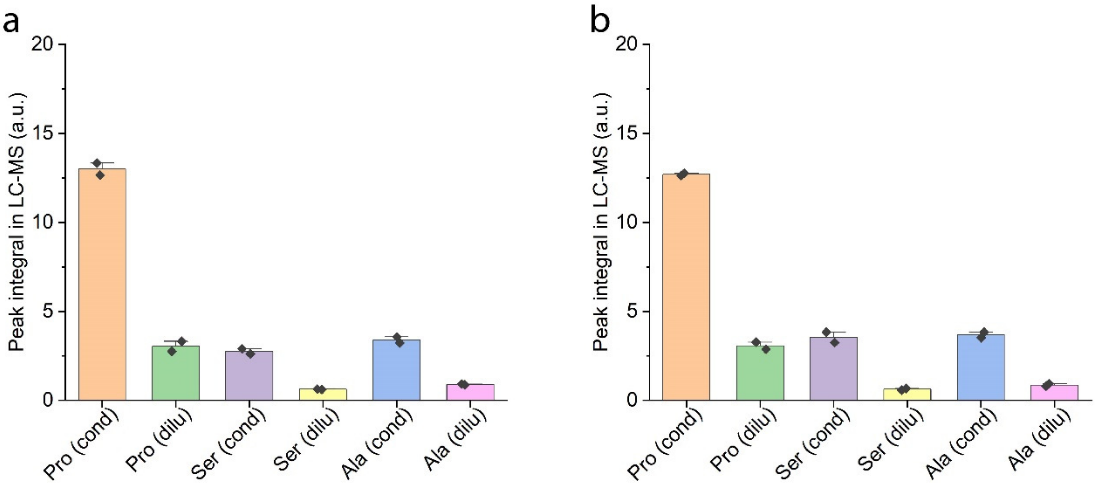

Supplementary Figure 11: LC-MS determination of amino acid concentrations in condensate and dilute phases for (a) NPM1-RNA condensates and (b) K72-ATP. Glycine was not detected on our LC-MS system. Data are expressed as mean ± standard deviation of n = 2 independent experiments.

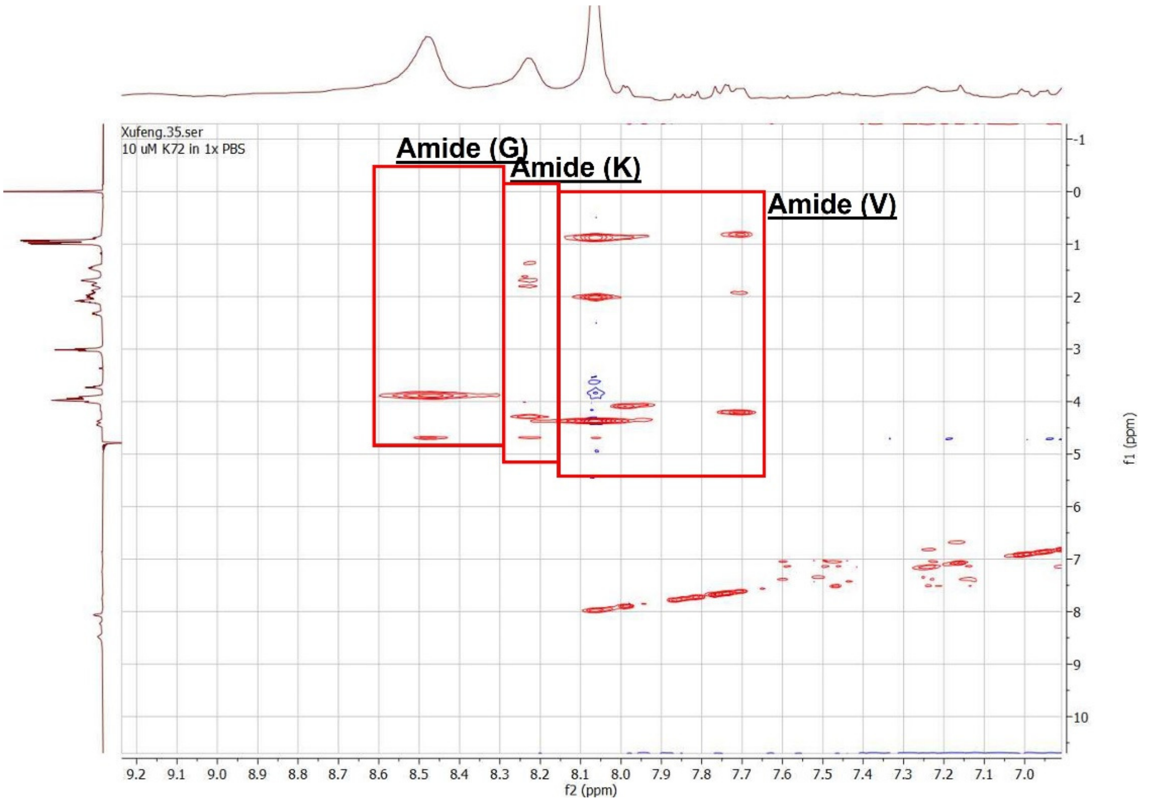

Supplementary Figure 12: TOCSY for the peak assignment of all the amide groups in K72.

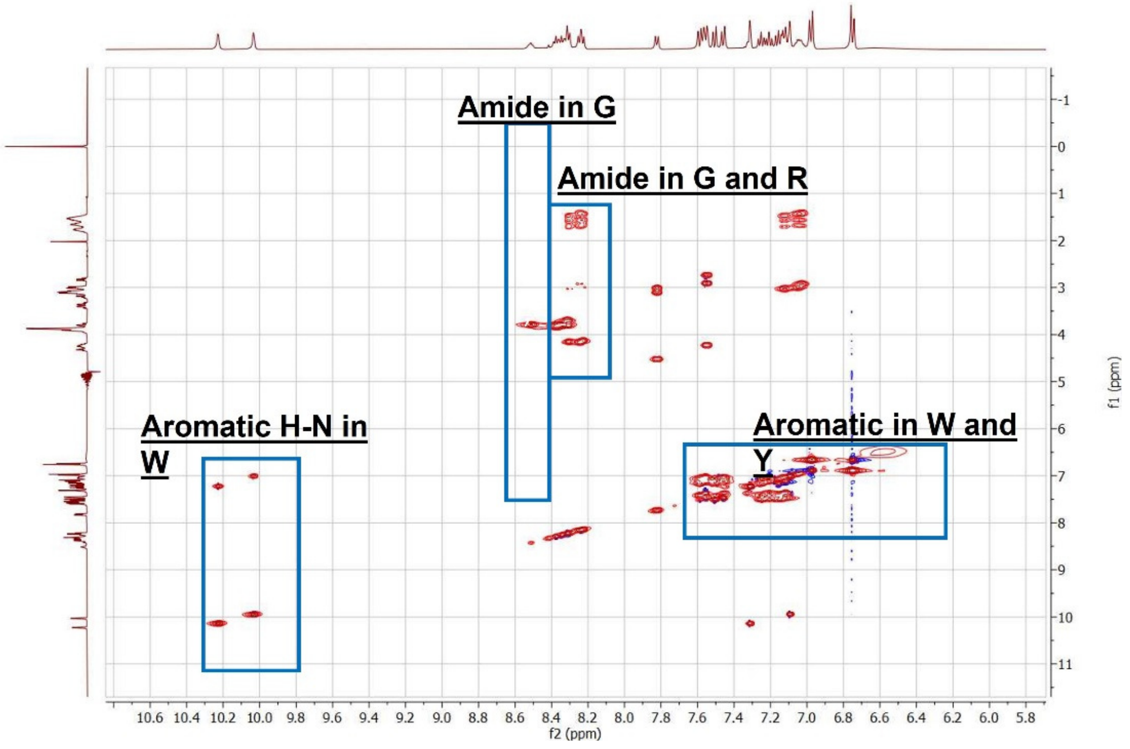

Supplementary Figure 13: TOCSY for the peak assignment of the aromatic and amide groups in WGR-4.

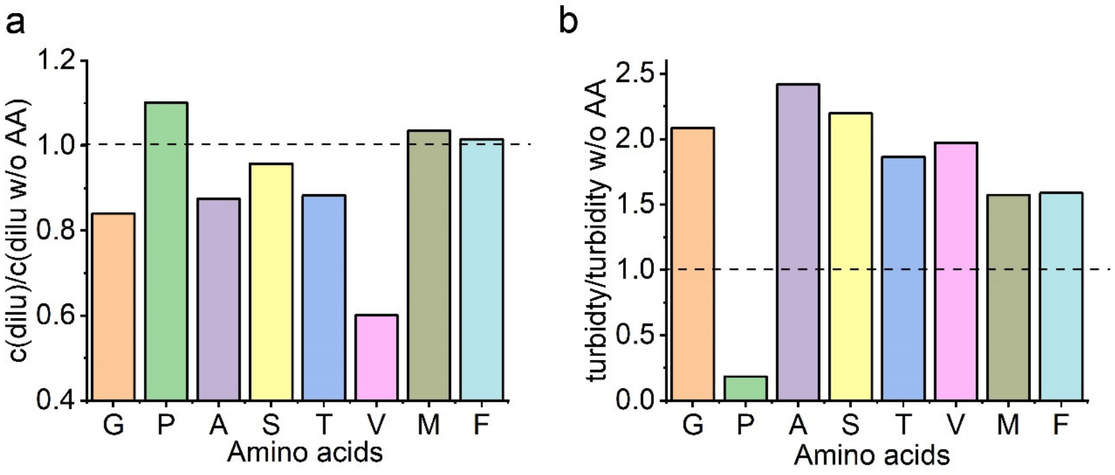

Supplementary Figure 14: The effects of AAs on (a) WGR-4 concentration in the dilute phase and (b) the turbidity of the whole solution for WGR-4 system. Data are expressed from n = 1 independent experiments.

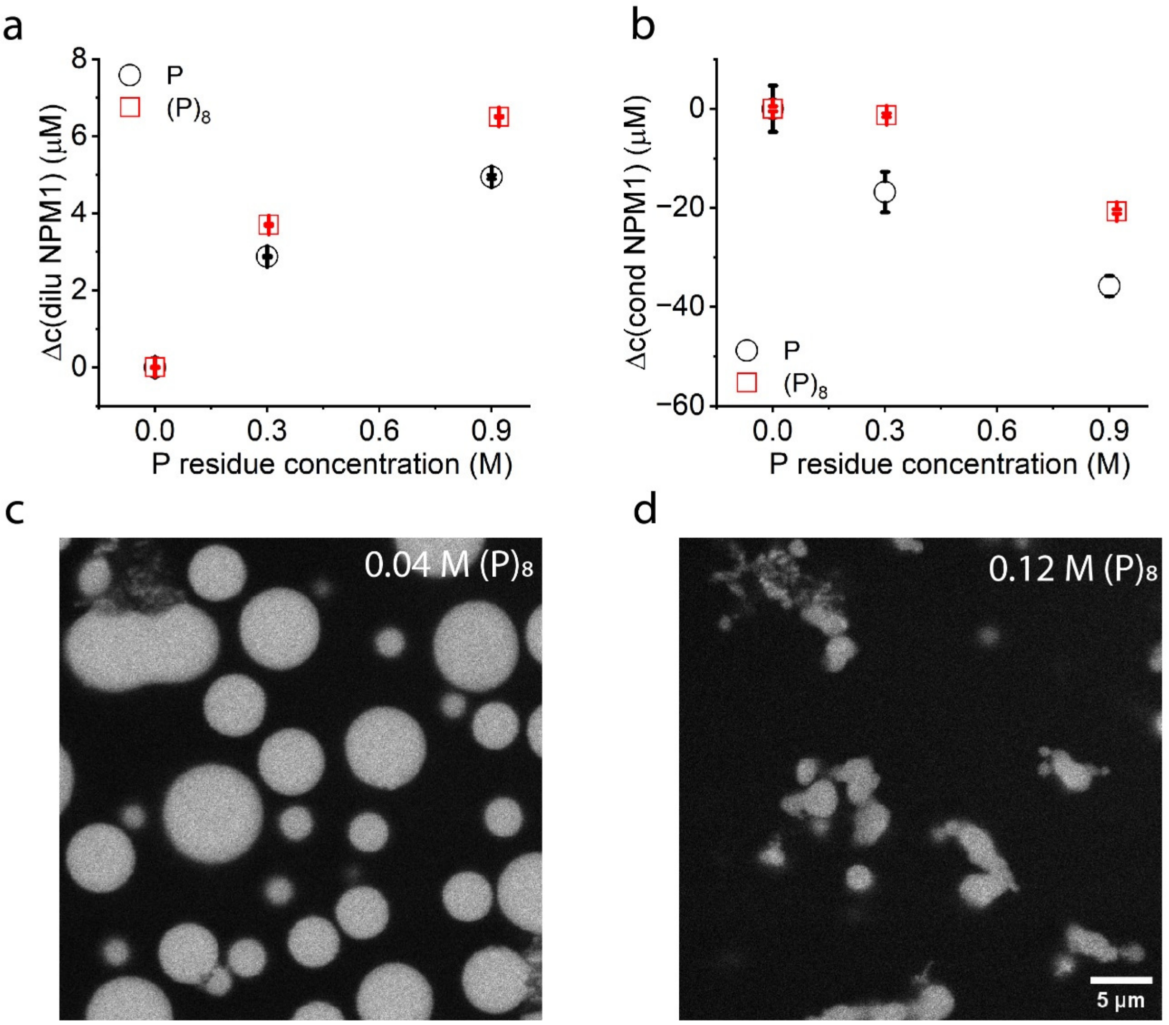

Supplementary Figure 15: (a) and (b). NPM1 concentration changes in the dilute and condensate phase after the addition of P and $(P)_8$ at the same ionic strength; (c) and (d).Fluorescence confocal microscopy images of NPM1-RNA condensates in NPM1-A488 channel after the addition of 0.04 and 0.12 M of $(P)_8$. Data are expressed as mean ± standard deviation of n = 3 independent experiments.

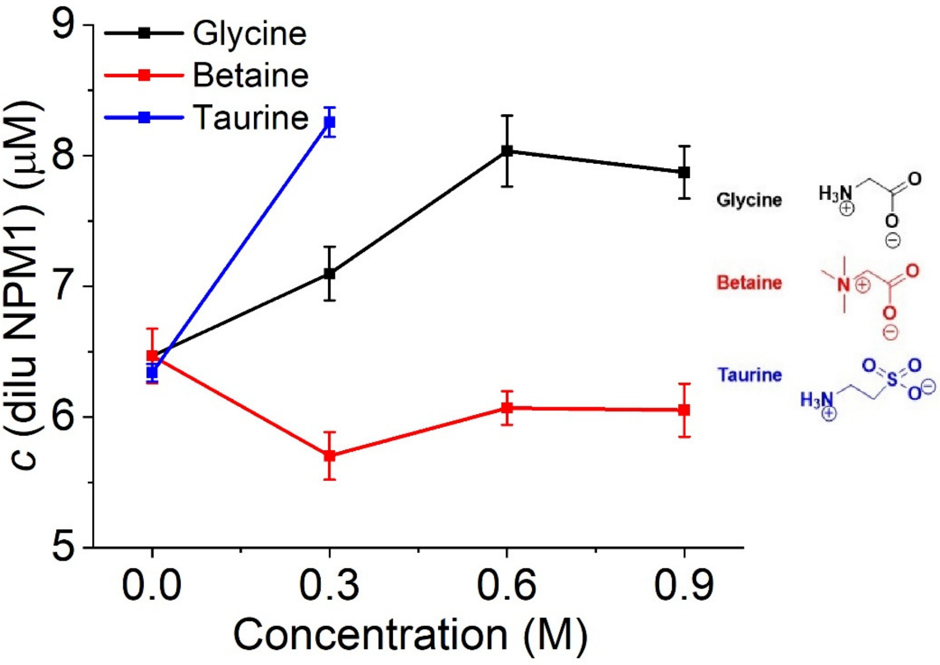

Supplementary Figure 16: NPM1 concentration change in the dilute phase for NPM1-RNA condensates as a function of concentration for glycine, betaine, and taurine (chemical structures shown on the right to the plot). Data are expressed as mean ± standard deviation of n = 3 independent experiments.

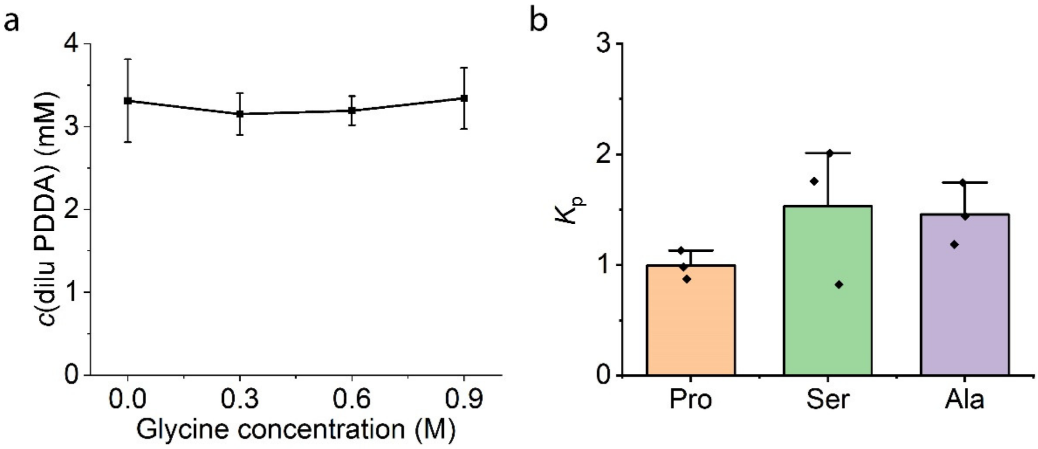

Supplementary Figure 17: (a) PDDA concentration change in the dilute phase for PDDA-PAA condensates as a function of concentration for glycine; (b) the measured partition coefficients (K_p) by NMR for three representative AAs (proline, serine, and alanine) in the condensate phase of PDDA-PAA systems. Data are expressed as mean ± standard deviation of n = 3 independent experiments.

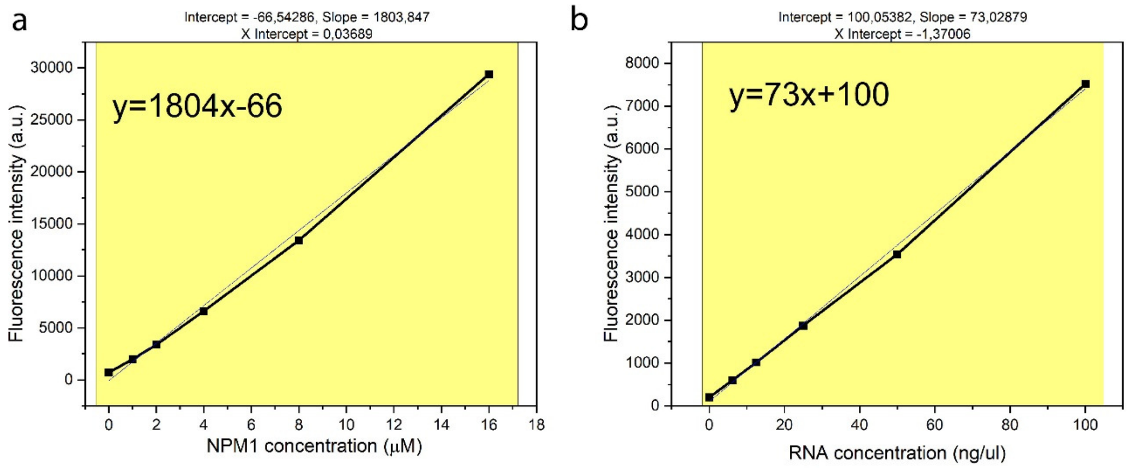

Supplementary Figure 18: Calibration curves for calculating the NPM1 and RNA concentrations in the dilute phase by fluorescence intensity measurement in the plate reader (Tecan Spark M10, 485/535 nm for NPM1-A488 and 620/680 nm for RNA-A647). The fitting equations are also displayed alongside the fitted calibration curves. Data are expressed from n = 1 independent experiments.

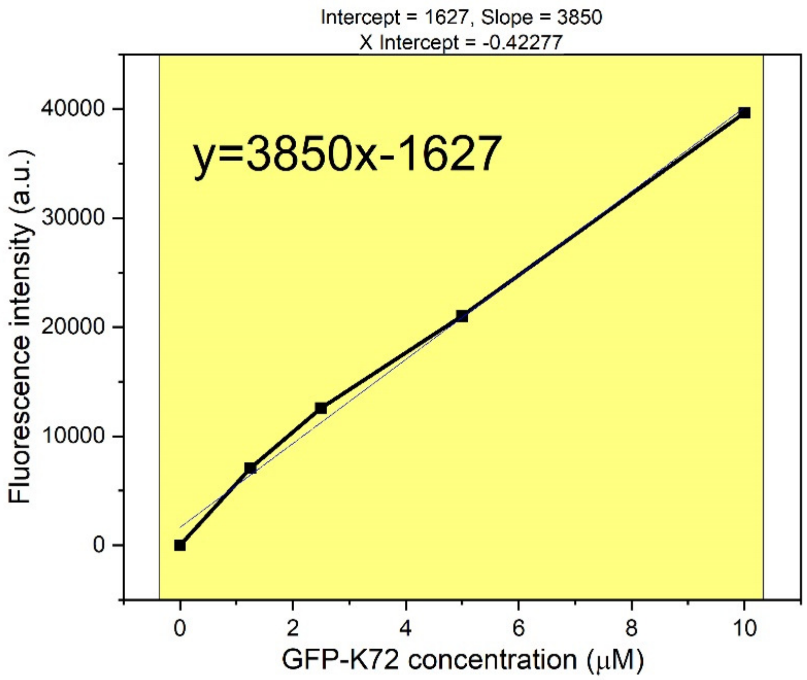

Supplementary Figure S19: Calibration curves for calculating the GFP-K72 concentrations in the dilute phase by fluorescence intensity measurement in the plate reader (Tecan Spark M10, 485/535 nm for GFP-K72). The fitting equation is also displayed alongside the fitted calibration curve. Data are expressed from n = 1 independent experiments.

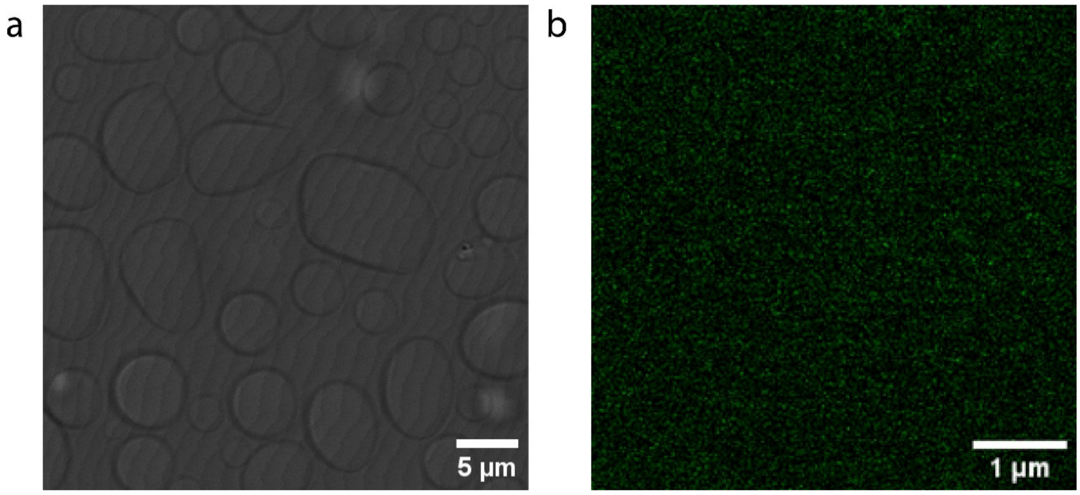

Supplementary Figure 20: (a) A representative brightfield image used to identify droplets larger than 5 µm in diameter (line patterns are artifacts in the transmitted light channel); b. After selecting an appropriate droplet, the field of view was zoomed in from the center of the droplet for image acquisition. Shown here is an example fluorescence frame used for RICS analysis.

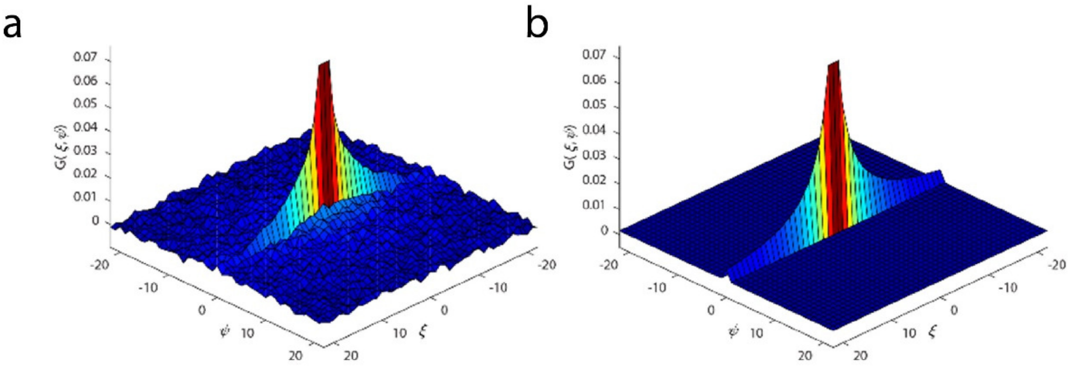

Supplementary Figure 21: a. the measured autocorrelation curve and b.the fitted autocorrelation curve for Figure 3. The fitting yielded a diffusion coefficient of 48 µm²/s.

Supplementary Table 1: pH measurement of Britton–Robinson (BR) buffer for FFssFF after the addition of glycine.

**pH of buffer**	**pH after addition of 0.3 M G**	**pH after addition of 1 M G**
6.23	6.13	6.00
6.53	6.44	6.29
6.78	6.69	6.53
7.08	6.97	6.79
7.37	7.25	7.04
7.69	7.50	7.26
7.99	7.71	7.43
8.36	7.94	7.62
